# Detection of *MPL*W515L/K Mutations and Determination of Allele Frequencies with a Single-Tube PCR Assay

**DOI:** 10.1371/journal.pone.0104958

**Published:** 2014-08-21

**Authors:** Hiraku Takei, Soji Morishita, Marito Araki, Yoko Edahiro, Yoshitaka Sunami, Yumi Hironaka, Naohiro Noda, Yuji Sekiguchi, Satoshi Tsuneda, Akimichi Ohsaka, Norio Komatsu

**Affiliations:** 1 Department of Hematology, Juntendo University School of Medicine, Tokyo, Japan; 2 Department of Life Science and Medical Bioscience, Waseda University, Tokyo, Japan; 3 Department of Transfusion Medicine and Stem Cell Regulation, Juntendo University Graduate School of Medicine, Tokyo, Japan; 4 Biomedical Research Institute, National Institute of Advanced Industrial Science and Technology (AIST), Ibaraki, Japan; Centro de Investigación Príncipe Felipe – CIPF, Spain

## Abstract

A gain-of-function mutation in the *myeloproliferative leukemia virus* (*MPL*) gene, which encodes the thrombopoietin receptor, has been identified in patients with essential thrombocythemia and primary myelofibrosis, subgroups of classic myeloproliferative neoplasms (MPNs). The presence of *MPL* gene mutations is a critical diagnostic criterion for these diseases. Here, we developed a rapid, simple, and cost-effective method of detecting two major *MPL* mutations, *MPL*W515L/K, in a single PCR assay; we termed this method DARMS (dual amplification refractory mutation system)-PCR. DARMS-PCR is designed to produce three different PCR products corresponding to *MPL*W515L, *MPL*W515K, and all *MPL* alleles. The amplicons are later detected and quantified using a capillary sequencer to determine the relative frequencies of the mutant and wild-type alleles. Applying DARMS-PCR to human specimens, we successfully identified *MPL* mutations in MPN patients, with the exception of patients bearing mutant allele frequencies below the detection limit (5%) of this method. The *MPL* mutant allele frequencies determined using DARMS-PCR correlated strongly with the values determined using deep sequencing. Thus, we demonstrated the potential of DARMS-PCR to detect *MPL* mutations and determine the allele frequencies in a timely and cost-effective manner.

## Introduction

Detecting clonal mutations and measuring mutant allele frequencies in malignant tissues are important tasks in clinical laboratories. In patients with essential thrombocytosis (ET) and primary myelofibrosis (PMF), acquired mutations in genes such as *JAK2* tyrosine kinase, *calreticulin* (*CALR*), and *myeloproliferative leukemia virus* (*MPL*) have been described [1]–[3]. *JAK2* and *MPL* mutations have been demonstrated to play causal roles in MPN development in animal models [4], [5]. Based on these findings, the identification of clonal mutations was deemed a diagnostic criterion for MPN by the World Health Organization (WHO) in 2008.

The major JAK2 mutation found in MPN patients is *JAK2*V617F (G1849T); therefore, epidemiological studies of MPN examining *JAK2*V617F have been greatly facilitated by PCR-based assays, such as ARMS (amplification refractory mutation system)-PCR [6], ABC (alternately binding probe competitive)-PCR [7], and allele specific (AS)-qPCR [8]–[11]. In contrast, a gain-of-function mutation in *MPL*, which encodes the thrombopoietin (TPO) receptor, has been identified in multiple sites within exon 10, making its detection with a simple PCR-based assay difficult. *MPL* mutations in MPN patients have traditionally been characterized using melting curve assays combined with Sanger sequencing [12], [13], quantitative-PCR [14], [15], or, more recently, deep sequencing [16], [17]. However, these assays generally require expensive machines and reagents and greater workloads. Partly because of the absence of a practical method for detecting *MPL* mutations, there have been many more epidemiological studies of MPN patients carrying the *JAK2* V617F mutation compared with *MPL* mutations.

Thus far, 10 types of *MPL* mutations have been identified in or around the transmembrane domain of MPL [17]–[19], and most of these result in the activation of MPL, even in the absence of TPO, and the subsequent activation of downstream targets [5], [20], [21]. These mutations include G1544T and TG1543_1544AA, which result in the substitution of tryptophan with leucine (W515L) or lysine (W515K), respectively, and are found in nearly three-quarters of patients bearing *MPL* mutations [17]. *MPL*W515L/K has been shown to promote tumorigenesis *in vivo* [5], and a higher mutant allele frequency is associated with progression to myelofibrosis [17]. Thus, detecting *MPL*W515L/K mutations and determining the relative ratios of W515L or W515K to the wild-type allele is critical for improving our understanding of MPN pathogenesis. In this work, we developed a cost-effective and practical method for simultaneously detecting both the *MPL*W515L and W515K mutations with high sensitivity and for determining the *MPL*W515L/K allele frequencies using a single PCR assay.

## Materials and Methods

### Standard DNA preparation

A 1,331-base-pair (bp) fragment containing the human *MPL* sequence (43348414 to 43349744 of NC_000001.11) was PCR-amplified from human genomic DNA (#G3048 Promega, Dane Country, USA) with a set of primers (forward primer: AAATCTGGCATCCTCTGCAGCATGAGTATTATTTG; reverse primer: CAAGAGGTTCTGTTTCAGTGAGTCAGGTCGTGT). The PCR product was subcloned into the pSP73 cloning vector (Promega) to generate pSP73/*MPL*-WT. An *MPL*W515L (G1544T) or W515K (TG1543_1544AA) mutation was introduced into pSP73/*MPL*-WT using the QuikChange Lightning Site-Directed Mutagenesis Kit (Agilent Technologies, Santa Clara, USA) according to the manufacturer's instructions. The sequences of all of the plasmids were confirmed via Sanger sequencing. A 3,339-bp plasmid carrying the wild-type *MPL*, W515L, or W515K DNA sequence was linearized by *Sca*I restriction enzyme digestion, subjected to agarose gel electrophoresis, and purified with the QIAquick gel extraction kit (Qiagen, Hilden, Germany). The purified DNA was mixed at the indicated ratio (see Results and Discussion) to create a standard template for dual amplification refractory mutation system (DARMS)-PCR.

### DARMS-PCR

**Figure 1:**
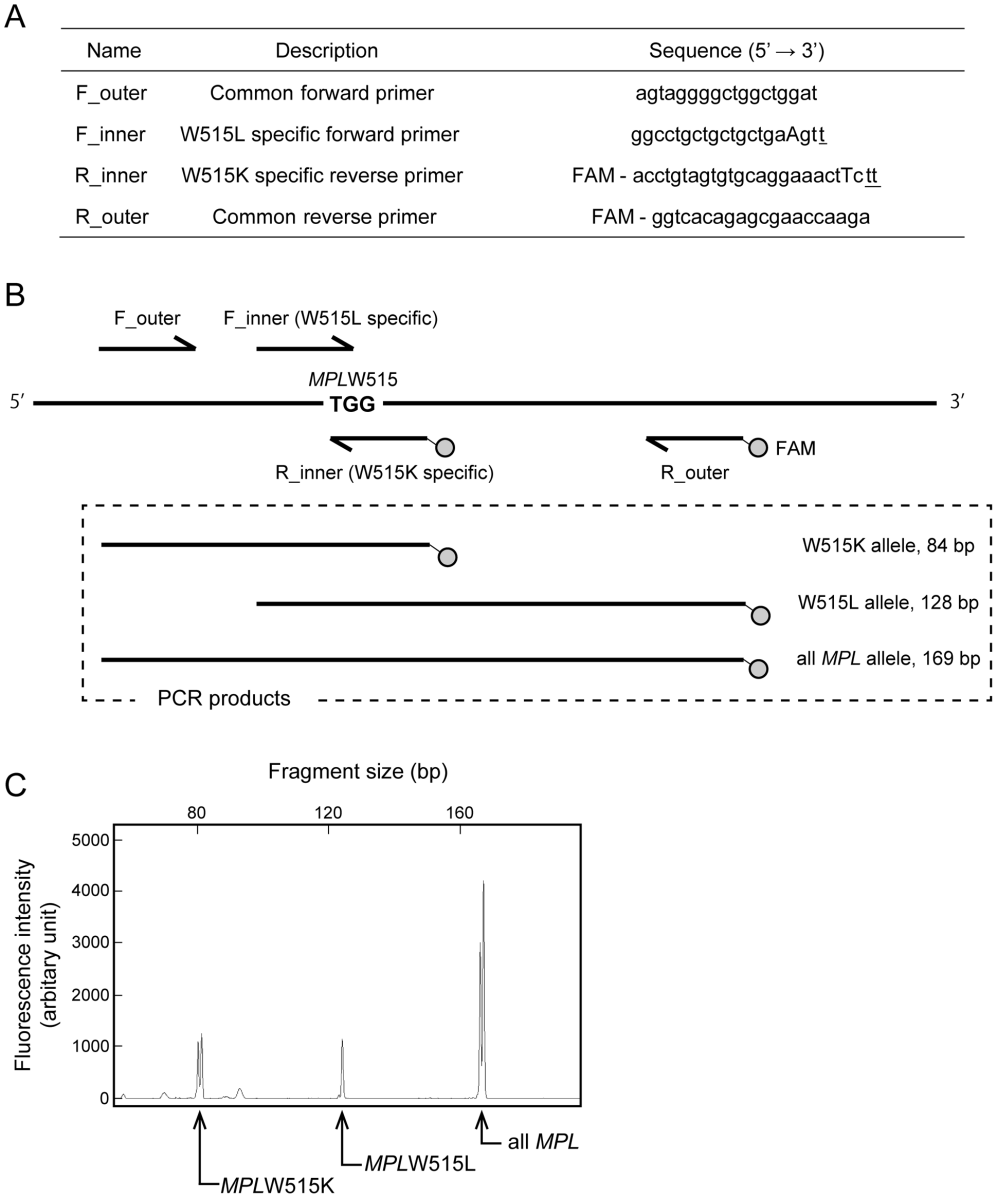
Detection of *MPL*W515L/K mutations using DARMS-PCR. (A) The primers used in the DARMS-PCR assay. The two inner primers harbored sequences (underlined) that matched *MPL*W515L or W515K, but not the wild-type allele. Other mismatches (capital letters) were introduced into the inner primers to reduce the annealing of the mutant-specific primers to the wild-type sequence. The reverse primers were labeled with FAM (5-carboxyfluorescein hydrate) at the 5′ terminus. (B) A schematic representation of DARMS-PCR products. The two outer primers were designed to generate a 169-base-pair (bp) PCR product from all *MPL* alleles. The F_inner and R_inner primers annealed specifically to the *MPL*W515L and W515K alleles, respectively; in combination with the outer primers, they generated 84- and 128-bp PCR products, respectively. From a mutant allele, both 169-bp and 84- or 128-bp fragments were amplified, while, only the 169-bp fragment was generated from the wild-type allele. (C) Demonstration of DARMS-PCR. A capillary electropherogram of DARMS-PCR products showing three peaks derived from wild-type *MPL*, W515L, and W515K. This result was obtained when PCR was performed with a standard DNA mixture containing equal ratios of *MPL* wild-type, W515L, and W515K alleles with a total copy number of 10^5^. The horizontal axis represents the fragment length, and the vertical axis represents the fluorescence intensity.

*MPL* sequences, including TGG1543_1545 (encoding wild-type W515), were amplified via ARMS-PCR [6] with the following modifications. The reaction mixture (20 µL) was composed of 1X AmpliTaq Gold Master Mix (Life Technologies), 120 nM of two outer primers (F_outer and R_outer), 1 µM of F_inner primer, 300 nM of R_inner primer, and template DNA (genomic DNA ranging from 19 to 253 ng). Note that with DARMS-PCR, the frequency of a given *MPL* mutant allele is determined by comparing the relative signal peak values of that mutant and all *MPL* alleles; thus, in theory, the template copy number can be variable. Indeed, we confirmed that using the abovementioned range of standard DNA template, the resulting mutant frequencies did not differ significantly from each other (data not shown). The primer sequences and positions are presented in Figure 1A and B. The PCR conditions were as follows: an initial denaturation at 95°C for 10 min; 28 cycles of denaturation at 95°C for 30 sec, annealing at 62°C for 30 sec, and extension at 72°C for 30 sec; and a final extension at 72°C for 7 min.

### Detection of MPL mutant alleles and determination of allele frequencies

DARMS-PCR products were analyzed via capillary electrophoresis. A capillary electrophoresis mixture was created that contained 1.0 µL of six-fold diluted DARMS-PCR products, 0.2 µL of GeneScan 500 LIZ Size Standard (Life Technologies, Carlsbad, USA), and 15.8 µL of Hi-Di formaldehyde (Life Technologies). A 17-µL aliquot of the mixture was heated at 86°C for 3 min, cooled on ice, and loaded onto an ABI 3130xl Genetic Analyzer (Life Technologies). The sizes of the PCR products were determined based on the size standard. The height of each fluorescence peak corresponding to wild-type *MPL*, W515K, or W515L was measured. The fluorescence peak values of a standard DNA mixture containing a known wild-type-to-mutant *MPL* ratio were used to generate the following formulas for calculating the *MPL* mutant allele frequencies (see Results and Discussion).

### Human specimens

Genomic DNA was purified from peripheral blood collected from 20 patients previously diagnosed with MPN at Juntendo University Hospital (Hongo, Tokyo, Japan) or other participating institutions [22]. This study was conducted in accordance with the Declaration of Helsinki and was approved by the ethics committee of Juntendo University School of Medicine (IRB#21076). Written informed consent for the use of samples and clinical records was obtained from all the patients prior to sample collection. Genomic DNA was purified from 200 µL of each blood sample using the QIAamp DNA Mini Kit (Qiagen). The genomic DNA concentrations were measured with the NanoDrop Lite spectrophotometer (Thermo Scientific, Waltham, USA), and the DNA was stored at −80°C until use.

### Targeted deep sequencing

A 169-bp fragment containing the human *MPL* sequence (43349278 to 43349446 of NC_000001.11) was PCR-amplified from genomic or standard plasmid DNA using forward (5′-AGTAGGGGCTGGCTGGAT-3′) and reverse (5′-GGTCACAGAGCGAACCAAGA-3′) primers (see above). The PCR products were subjected to agarose gel electrophoresis and were purified with the QIAquick gel extraction kit (Qiagen). Sample libraries were prepared with the TruSeq DNA LT Sample Prep Kit (Illumina, San Diego, USA) according to the manufacturer's instructions, and the libraries were deep sequenced with a MiSeq benchtop sequencer (Illumina). The data analysis was performed using CLC Genomics Workbench software version 6.5 (CLC Bio, Aarhus, Denmark) with a minimum coverage of 150,000 and a minimum variant frequency of 1%. The mutant allele frequency was calculated by dividing the number of mutant sequence reads by the sum of the mutant and wild-type reads.

## Results and Discussion

### Establishment of DARMS-PCR

To develop a single PCR assay for detecting the two major mutant alleles, *MPL*W515L (G1544T) and W515K (TG1543_1544AA), we modified an ARMS-PCR method [6] by adding a total of four primers to each reaction; we renamed the method DARMS-PCR. Two mutant-specific primers (F_inner and R_inner) and two common primers (F_outer and R_outer) were designed (Figure 1A and B). *MPL*G1544T, TG1543_1544AA, and a control fragment for all *MPL* alleles generated unique PCR products that were 128, 84, and 169 bp in size, respectively (Figure 1B). The two reverse primers (R_inner and R_outer) were labeled with the fluorescent dye FAM (5-carboxyfluorescein hydrate), which allowed us to determine the lengths and quantity of the PCR products using a capillary sequencer. According to the design of the assay, when a template containing a mixture of purified DNA fragments representing *MPL*G1544T, TG1543_1544AA, and wild-type alleles was amplified under optimized conditions (see Materials and Methods), three PCR products corresponding to the different alleles were detected (Figure 1C). All three PCR products produced dual peaks, presumably because of an indefinite adenine addition at the end of each amplicon by Taq polymerase (Figure 1C). The sum of the peak height values from each dual peak was used in the following analyses.

### Determination of *MPL* mutant allele frequency using DARMS-PCR

**Figure 2:**
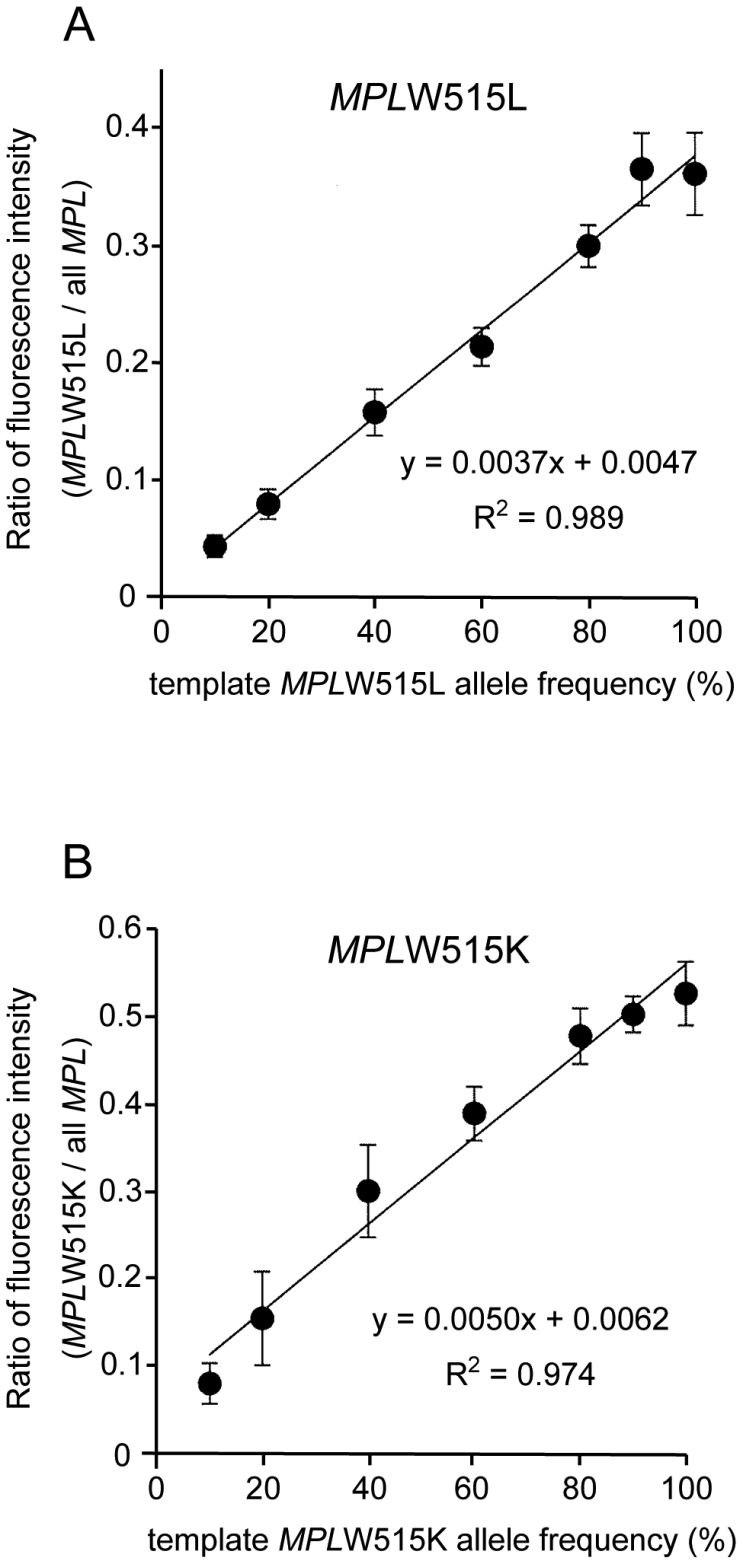
Demonstration of DARMS-PCR to determine *MPL* mutant allele frequencies. Standard curves for the quantification of *MPL*W515L (A) and W515K (B) allele frequencies. A series of templates containing different amounts of mutant allele DNA were used for DARMS-PCR. The fluorescence peak value of the mutant allele over the value for all *MPL* alleles was calculated for each template. The mean values and errors obtained from three independent experiments are shown.

To examine whether the *MPL* mutant allele frequency could be quantitatively determined using DARMS-PCR, we created a series of standard DNA templates containing different amounts of wild-type and mutant alleles. Templates containing 10, 20, 40, 60, 80, 90, or 100% mutant (W515L or W515K) allele DNA were used for DARMS-PCR, and the fluorescent intensities of the products were measured with the capillary sequencer (see Materials and Methods). As shown in Figure 2A and B, the fluorescence intensity of the mutant alleles over the intensity of all *MPL* alleles demonstrated a nearly linear correlation, indicating the accuracy of DARMS-PCR for quantitatively determining *MPL* mutant allele frequencies. Based on this result, we generated the following formulas to calculate the *MPL* mutant allele frequencies: *MPL*W515L allele frequency (%) = 270×[fluorescence value (arbitrary unit) for *MPL*W515L PCR product]/[fluorescence value (arbitrary unit) for all *MPL* allele PCR products]; and *MPL*W515K allele frequency (%) = 200×[fluorescence value (arbitrary unit) for *MPL*W515K PCR product]/[fluorescence value (arbitrary unit) for all *MPL* allele PCR products].

### Detection limit of *MPL* mutations using DARMS-PCR

**Figure 3:**
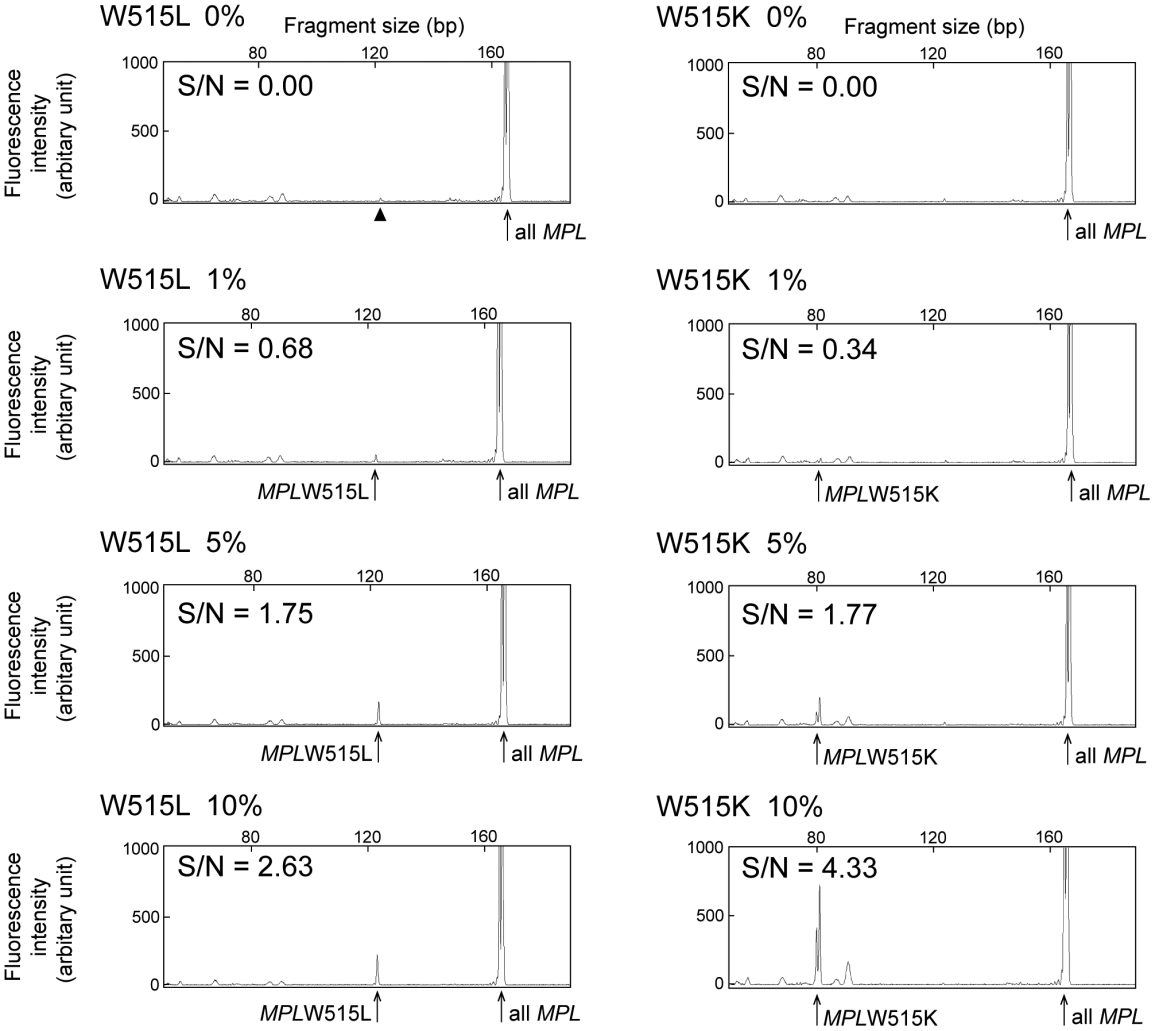
Lower limit of *MPL* mutation detection by DARMS-PCR. Capillary electropherograms for DARMS-PCR assays with low levels of *MPL*W515L or W515K mutant alleles are presented. The average fluorescence intensities for nonspecific PCR products were defined as noise. S/N ratios, which were calculated as the fluorescence peak value for a mutant PCR product over the value for noise, are shown. An arrow head in the W515L 0% panel indicates a false PCR product (see Results and Discussion).

We then examined the lower detection limit of DARMS-PCR under the conditions used for determining the mutant allele frequencies. We performed DARMS-PCR with DNA containing *MPL* mutant frequencies of 0, 1, 5, and 10%, and we obtained peak heights of 0, 55, 165, and 221 for *MPL*W515L and 0, 33, 199, and 731 for W515K (Figure 3). We then determined the average peak heights for nonspecific PCR products, which were defined as noise. The signal-to-noise (S/N) ratio was calculated as the fluorescence value for the peak of the mutant allele divided by the value for noise (Figure 3). When the allele frequency was 5%, the S/N ratios were 1.75 and 1.77 for *MPL*W515L and W515K, respectively. Thus, we concluded that the detection limit of DARMS-PCR was greater than 5% for both the *MPL*W515L and 515K mutations.

### Validation of the DARMS-PCR assay results using deep sequencing

**Figure 4:**
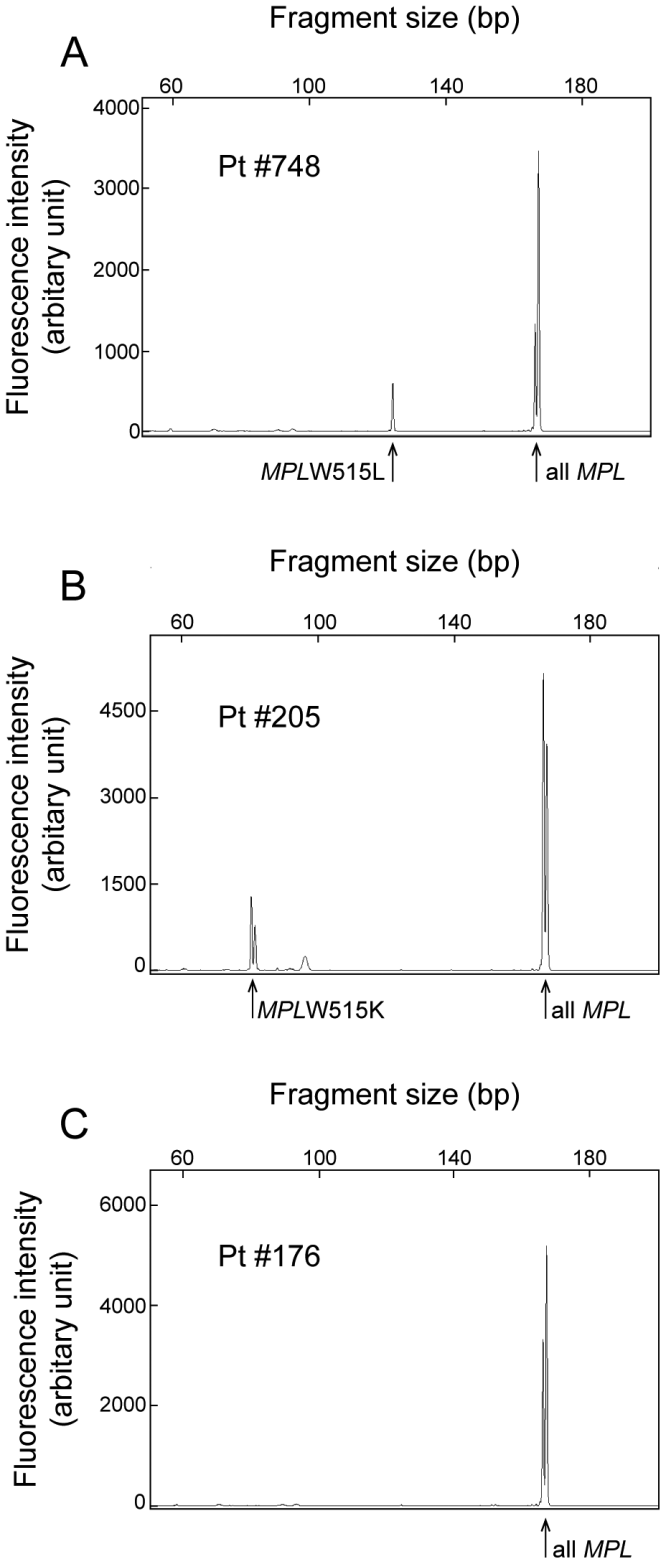
Detection of *MPL* mutations in human specimens. Representative capillary electropherograms from the DARMS-PCR assay with human specimens are presented. Patients #748 (A), #205 (B), and #176 (C) harbored *MPL*W515L, W515K, and wild-type alleles, respectively. The calculated allele frequencies are presented in Table 1.

**Figure 5:**
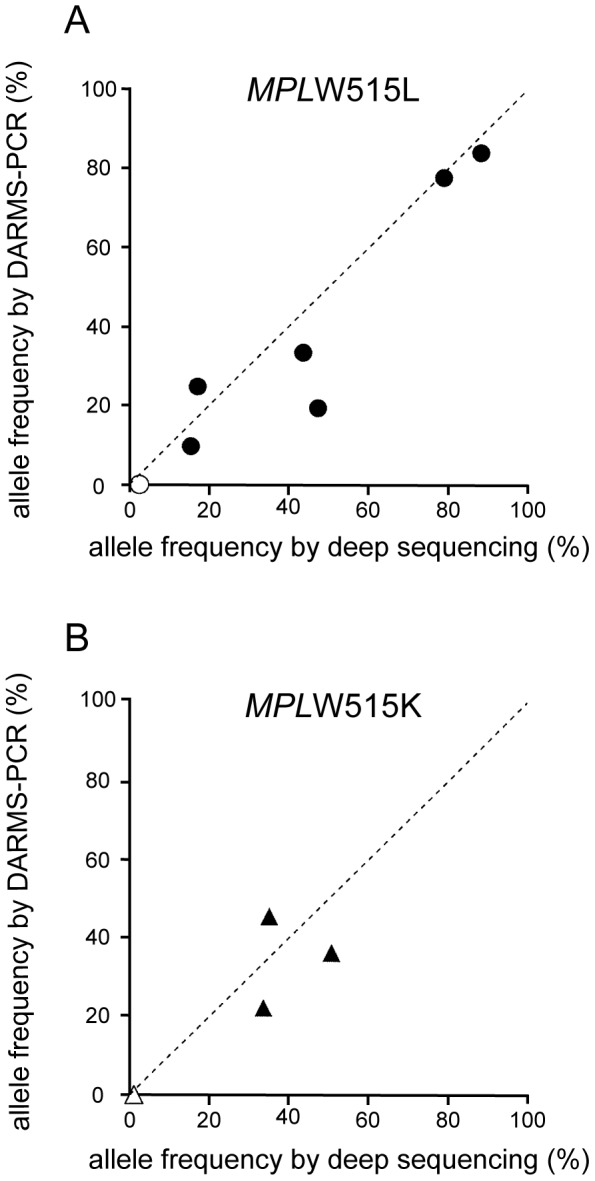
Comparison of *MPL* mutant frequencies determined using DARMS-PCR and deep sequencing. The horizontal axis represents the frequencies of the *MPL*W515L (A) or W515K (B) mutant alleles determined using deep sequencing, and the vertical axis represents the mutant allele frequencies determined using DARMS-PCR. The specific values are listed in Table 1. Filled symbols represent alleles identified using both DARMS-PCR and deep sequencing. Open symbols represent alleles identified only using deep sequencing.

To examine the accuracy of DARMS-PCR for evaluating human specimens, we analyzed genomic DNA samples from 20 MPN patients whose *MPL* mutation status and allele frequencies had been determined using deep sequencing (see Materials and Methods). The formulas defined in Figure 2 were used to calculate the *MPL* mutant allele frequencies from the fluorescence peak values, which were determined using DARMS-PCR (Figure 4, Table 1). The results indicated that, using DARMS-PCR, 6 *MPL*W515L-positive and 3 W515K-positive MPN patients were successfully identified. Patient #120 was not identified due to having an *MPL* mutant frequency of 1.0% (according to deep sequencing), which is below the DARMS-PCR detection limit (5%) (Table 1). Patient #127, who was initially identified as positive for *MPL*W515K using DARMS-PCR, was later found to be positive for both *MPL*W515L and W515K using deep sequencing (Table 1). The mutant allele frequency of *MPL*W515L in this patient was 2.5%, which was below the detection limit of DARMS-PCR. The remaining 10 patients, who were identified as wild-type for *MPL* using deep sequencing, had *MPL* allele frequencies below the positivity threshold of DARMS-PCR and thus were determined to be negative. Overall, although DARMS-PCR had a limitation of detection of mutant allele frequencies lower than 5%, all the patient specimens identified as having *MPL* mutations using DARMS-PCR were confirmed using deep sequencing. There was a strong correlation between the *MPL* mutant frequencies determined using DARMS-PCR versus deep sequencing, with errors ranging from −28.0% to 10.4% (Figure 5, Table 1). Further research is required to evaluate the quantitative nature of DARMS-PCR. Nevertheless, these data imply that DARMS-PCR can be used as a diagnostic tool to identify *MPL* mutations in MPN patients.

**Table 1:** *MPL* allele frequencies determined using DARMS-PCR and deep sequencing.

		*MPL*W515L allele frequency (%)*		*MPL*W515K allele frequency (%)*	
*MPL* allele	Patientno.	DARMS-PCR	Deep sequencing	DARMS-PCR	Deep sequencing
*MPL*W515L	146	77.8	79.0	N.D.	0.0
	177	84.1	88.4	1.8	0.0
	526	24.7	17.1	N.D.	0.2
	748	33.4	43.7	N.D.	0.0
	782	19.4	47.4	0.9	0.0
	830	9.7	15.4	0.4	0.0
*MPL*W515L/K	127	N.D.	2.5	36.3	50.7
*MPL*W515K	120	N.D.	0.1	N.D.	1.0
	193	N.D.	0.1	22.2	33.6
	205	N.D.	0.1	45.4	35.0
Wild-type *MPL*	6	N.D.	0.0	N.D.	0.0
	50	N.D.	0.0	N.D.	0.1
	158	1.8	0.2	N.D.	0.0
	160	N.D.	0.0	0.5	0.0
	176	N.D.	0.6	N.D.	0.0
	185	N.D.	0.3	0.8	0.0
	199	N.D.	0.0	0.6	0.5
	607	0.7	0.0	N.D.	0.0
	688	1.0	0.1	N.D.	0.0
	710	N.D.	0.2	0.4	0.0

*Allele frequencies below 1%, as determined using deep sequencing, were defined as negative for the assessed mutation. Note that allele frequencies below 5%, as determined using DARMS-PCR, were also defined as negative based on the results in Figure 3. N.D.: the corresponding peak was not detected.

As observed with the standard template without mutant alleles (shown by the arrowhead at the W515L 0% panel in Figure 3) and with patient specimens that were defined as negative for *MPL*W515K or W515L with deep sequencing (Table 1), the DARMS-PCR generates false-positive PCR products and thus the subsequent appearance of low but clear allele frequencies. These false-positives presumably originate from faulty priming by inner primers that are capable of annealing to both mutant andwild-type alleles but are designed not to produce PCR products from the wild-type allele with introduced mutations (see Figure 1). Further optimization of the PCR program and primer sequences and positions is required to make the assay more sensitive and accurate. Although the deep-sequencer is apparently more accurate in terms of detecting the *MPL*W515K/L mutation, we observed potential error calls with less than 1% mutation frequency (see Table 1). This is likely the result of an erratic amplification during the sequencing sample preparation and/or a casual incident during the deep-sequencing. In addition, the current version of DARMS-PCR assesses only two major *MPL* mutations: *MPL*W515K/L, which account for approximately 75% of all MPL mutations [17]; thus, 25% of patients bearing rare *MPL* mutations, such as *MPL*W515S and *MPL*S505N [17], cannot be detected with this method. The incorporation of more primers into current DARMS-PCR and/or the establishment of more DARMS-PCR protocols for detecting other rare *MPL* mutations is required for more comprehensive assessment of *MPL* mutations.

In summary, we have developed and validated DARMS-PCR, a cost-effective, rapid, and accurate method for identifying two major *MPL* mutations with a single PCR assay. Although deep sequencing can be the most accurate method for detecting mutant alleles and measuring allele frequencies, it is considerably more expensive and time-consuming than DARMS-PCR. Compared with the specialized thermal cycler equipped with a fluorescence detection system that is needed to perform a melting curve analysis [12], [13], DARMS-PCR requires only a general thermal cycler and a capillary sequencer (or even an agarose gel electrophoresis apparatus). DARMS-PCR is unique in its ability to simultaneously detect two mutations in one single-tube PCR assay; moreover, this method can be applied to detect other mutations as well. By employing this assay, the identification of approximately 75% of MPN patients with *MPL* mutations should be possible (see Introduction); thus, the use of DARMS-PCR will enhance screening for patients with *MPL*W515L/K mutations and will deepen our understanding of the pathogenesis of MPN.
