# Correction: Detection of MPLW515L/K Mutations and Determination of Allele Frequencies with a Single-Tube PCR Assay

**DOI:** 10.1371/journal.pone.0124208

**Published:** 2015-04-01

**Authors:** The PLOS ONE Staff

There are errors in the first sentence of the “Targeted deep sequencing” section of the Materials and Methods. The correct sentence is: A 169-bp fragment containing the human MPL sequence (43349278 to 43349446 of NC_000001.11) was PCR-amplified from genomic or standard plasmid DNA using forward (5′- TGACCGCTCTGCATCTAGTGC -3′) and reverse (5′-GGTCACAGAGCGAACCAAGA-3′) primers (see above).

There is an error in Fig. 1. The first primer sequence in Fig. 1A is incorrect. Please view Fig. 1 here.

**Fig 1:**
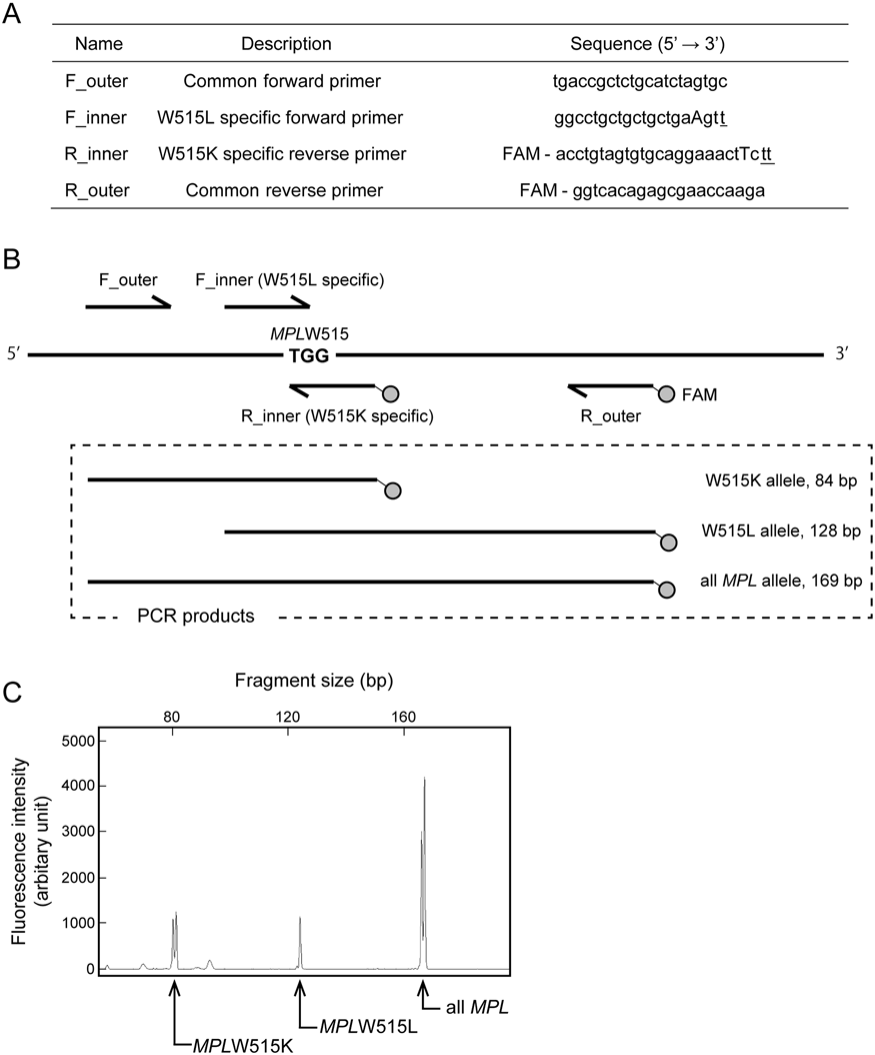
Detection of *MPL*W515L/K mutations using DARMS-PCR. (A) The primers used in the DARMS-PCR assay. The two inner primers harbored sequences (underlined) that matched *MPL*W515L or W515K, but not the wild-type allele. Other mismatches (capital letters) were introduced into the inner primers to reduce the annealing of the mutant-specific primers to the wild-type sequence. The reverse primers were labeled with FAM (5-carboxyfluorescein hydrate) at the 5′ terminus. (B) A schematic representation of DARMS-PCR products. The two outer primers were designed to generate a 169-base-pair (bp) PCR product from all *MPL* alleles. The F_inner and R_inner primers annealed specifically to the *MPL*W515L and W515K alleles, respectively; in combination with the outer primers, they generated 84- and 128-bp PCR products, respectively. From a mutant allele, both 169-bp and 84- or 128-bp fragments were amplified, while, only the 169-bp fragment was generated from the wild-type allele. (C) Demonstration of DARMS-PCR. A capillary electropherogram of DARMS-PCR products showing three peaks derived from wild-type *MPL*, W515L, and W515K. This result was obtained when PCR was performed with a standard DNA mixture containing equal ratios of *MPL* wild-type, W515L, and W515K alleles with a total copy number of 10^5^. The horizontal axis represents the fragment length, and the vertical axis represents the fluorescence intensity.

## References

[1] Takei H, Morishita S, Araki M, Edahiro Y, Sunami Y, Hironaka Y, et al (2014) Detection of *MPL*W515L/K Mutations and Determination of Allele Frequencies with a Single-Tube PCR Assay. PLoS ONE 9(8): e104958 doi: 10.1371/journal.pone.0104958 25144224 10.1371/journal.pone.0104958 PMC4140728

